# Ecological assembly of activated sludge microbiomes under contrasting process configurations and influent regimes

**DOI:** 10.3389/fbioe.2026.1912780

**Published:** 2026-09-21

**Authors:** Hongjing Luo, Chunhui Zhao, Sihui Yu, Chang Zhou, Daihua Wei, Weitai Lin, Xiaolei Wang, Zheng Gong, Kunpeng Xie

**Affiliations:** 1 Key Laboratory of Plant Biotechnology of Liaoning Province, School of Life Sciences, Liaoning Normal University, Dalian, China; 2 Zhejiang Andmir Biotechnology Co., Ltd, Hangzhou City, Zhejiang, China

**Keywords:** activated sludge, community assembly, influent composition, microbial communities, petrochemical wastewater treatment plants (PWWTPs), process configuration

## Abstract

Activated sludge microbial communities are central to pollutant removal and operational stability in petrochemical wastewater treatment plants (PWWTPs). However, in full-scale systems, the relative associations of process configuration and influent composition with microbial community organization and treatment performance remain difficult to disentangle. This study investigated four full-scale PWWTPs in Northeast China, including three oxic systems and one anoxic/oxic system receiving different proportions of petrochemical, mixed industrial, and domestic wastewater inputs. We combined 16S rRNA gene sequencing, phylogenetic bin-based null model analysis (iCAMP), and partial least squares path modeling (PLS-PM) to evaluate microbial community structure, inferred assembly patterns, and their associations with influent characteristics, effluent quality, and chemical oxygen demand (COD) removal. The oxic systems showed higher alpha diversity than the anoxic/oxic system, although this comparison was potentially influenced by differences in influent composition and site-specific operational conditions. Community composition differed among influent types, with Proteobacteria, Bacteroidota, Acidobacteriota, and Planctomycetota showing contrasting enrichment patterns. iCAMP indicated a predominance of stochastic assembly patterns in the oxic systems and deterministic assembly in the anoxic/oxic system. Core zOTUs, including members of Rhodocyclaceae, Pseudomonadaceae, and *Candidatus Berkiella*, were associated with COD and nitrogen removal, supporting their potential as ecological indicators of treatment performance. Within the PLS-PM, influent characteristics showed stronger direct statistical associations with COD removal than process configuration, whereas process-related associations were represented mainly by indirect paths through microbial community composition. These findings highlight the joint influence of influent composition and process configuration on activated sludge microbial communities and treatment performance while providing an ecological basis for future microbiome-informed monitoring and process diagnosis in PWWTPs.

## 1 Introduction

The petrochemical industry generates substantial quantities of petrochemical wastewater (PWW) containing diverse organic and inorganic constituents introduced during processing (Wei et al., 2019). Untreated PWW is difficult to manage because of its complex composition, high toxicity, and low biodegradability, and it can pose serious environmental risks (García-Sanchez et al., 2012). Therefore, petrochemical wastewater treatment plants (PWWTPs) commonly rely on combined physical, chemical, and biological processes to remove contaminants before discharge. Available treatment options include membrane separation, adsorption, coagulation, advanced oxidation, biological treatment, and integrated systems (Ben et al., 2018; Wei et al., 2019). Biological treatment is widely used because it can be efficient and cost-effective for large-scale wastewater management (Grandclément et al., 2017). In activated sludge systems, aerobic, anoxic, and anaerobic microbial consortia contribute to organic matter degradation and nutrient transformation (Komatsu et al., 2020). The structure and function of activated sludge microbial communities are therefore closely linked to treatment performance and process stability in industrial WWTPs (Lu et al., 2016).

Microbial community composition is influenced not only by process design but also by influent chemistry and loading (Chen et al., 2022). Chemical characteristics of incoming wastewater, including chemical oxygen demand, total nitrogen, and other pollutant loads, can strongly affect removal efficiency and microbial community structure (Li et al., 2022). Previous studies have identified distinct dominant bacteria in different WWTPs and petrochemical treatment systems (Behnami et al., 2018; Du et al., 2020; Kim et al., 2020). Community diversity and composition have also been linked to wastewater treatment stability (Jiang et al., 2021; Mielczarek et al., 2013). Researchers have developed statistical techniques to describe and compare the structure of these microbial communities (Hughes et al., 2001; Sogin et al., 2006). Statistical and ecological approaches now make it possible to compare microbial community structure and infer how deterministic processes, such as environmental filtering and biotic interactions, and stochastic processes, such as demographic fluctuation, dispersal, and drift, may jointly shape community assembly in engineered systems (Barberán et al., 2012; Lauber et al., 2009; Sun et al., 2020).

Jiang et al. (2021) investigated three types of petrochemical activated sludge using the beta nearest taxon index (βNTI) and a neutral model, showing that selection and stochasticity may both contribute to microbial community organization under industrial wastewater conditions. Kim et al. (2020) further monitored temporal variation in a full-scale petroleum refinery wastewater treatment plant and linked microbial dynamics to influent characteristics. Compared with whole-community neutral community models, the phylogenetic bin-based null model analysis iCAMP can partition taxa into phylogenetically related bins and estimate the relative contributions of selection, dispersal-related processes, and drift-like fractions within each bin (Ning et al., 2020). This bin-based strategy is useful for complex industrial systems because different lineages may respond differently to toxicity, redox conditions, and substrate availability, given the knowledge gap regarding the specific assembly mechanisms of iCAMP in unique environmental communities. We applied this approach to investigate the assembly mechanisms of the microbial communities in the PWWTPs, in order to provide new ideas for using microbial communities’ regulation to enhance PWW treatment efficiency.

The PWW contains various toxic and refractory organic pollutants that are difficult to eliminate (Jain et al., 2020). Moreover, the components and characteristics of different PWW streams (i.e., oil-containing wastewater, petroleum refinery wastewater, and mixed petroleum industrial wastewater) vary substantially. Microbial community diversity in WWTPs can be affected by influent composition (Wang et al., 2022), treatment compartments (e.g., anoxic and oxic tanks) (Chen et al., 2022; Wang et al., 2020), physicochemical parameters (pH and temperature) (Xu et al., 2017; Zhang et al., 2019), and operational parameters such as hydraulic retention time, aeration, sludge age, and reflux strategy (Maza-Márquez et al., 2019; Zhang et al., 2018). Because these variables frequently covary in full-scale facilities, it is challenging to independently attribute community differences to process configuration or influent composition without paired experimental designs (Wang C. et al., 2021).

In the current work, activated sludge samples were collected from four full-scale PWWTPs in an industrial park in Northeast China. The systems differed in both process configuration and influent composition, allowing us to describe field-scale covariation among wastewater characteristics, activated sludge microbial community structure, inferred assembly patterns, and pollutant removal. We used 16S rRNA gene sequencing, iCAMP, and PLS-PM to address the following questions: (1) How do microbial diversity and structure vary among the sampled influent compositions and process configurations? (2) Which core zOTUs are consistently detected, and how are they statistically associated with pollutant removal? (3) What null-model assembly patterns are inferred across the sampled systems? (4) Which measured influent, effluent, and microbial variables are most strongly associated with COD removal? Given the observational design, this study does not claim to fully disentangle process effects from influent and site-specific operational effects; instead, it provides field evidence and hypotheses for future paired and functionally validated studies.

## 2 Materials and methods

### 2.1 Wastewater and PWWTPs system

Activated sludge samples were collected from four PWWTP systems (P-94, P-320, P-440, and P-450) located in an industrial park in Northeast China, with treatment capacities of 1,200, 600, 300, and 600 tons h^-1^, respectively. Systems P-94, P-440, and P-450 operated as single oxic (O) configuration (Figure 1a), whereas system P-320 employed an anoxic/oxic (A/O) configuration (Figure 1b). Because microbial communities may differ between treatment stages within the A/O process, activated sludge was sampled separately from the anoxic tank and the oxic tank of system P-320. In contrast, the activated sludge samples were collected from the oxic tank of each O process (P-94, P-440, and P-450). The sampling locations, corresponding process configurations, and parameter information are summarized in Supplementary Table S1.

**FIGURE 1:**
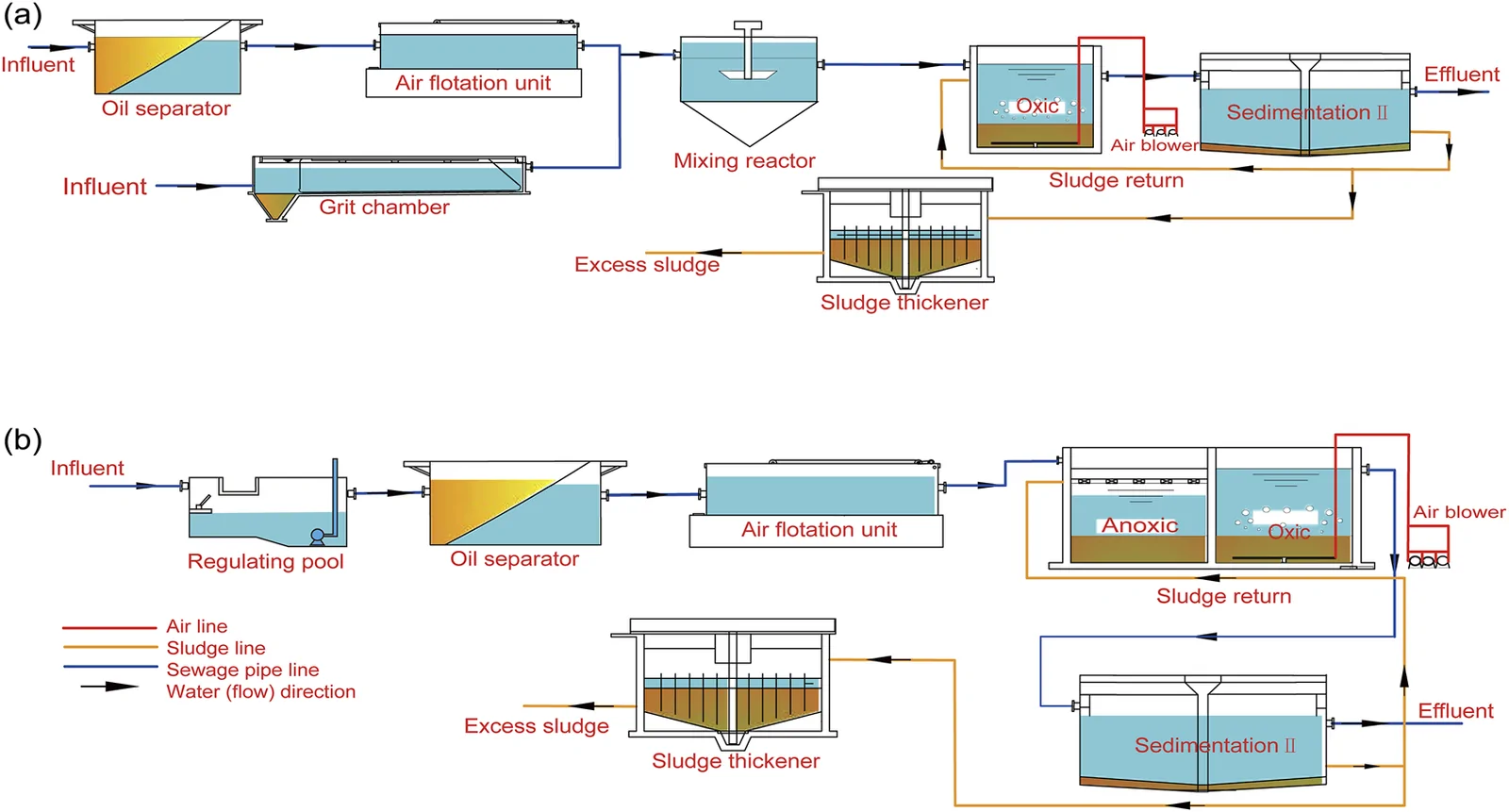
Schematic diagram of PWWTPs with sampled compartments. The primary treatment processes of the four wastewater treatment plants included an oil-water separator, a mixing reactor, an air flotation unit, an oxic tank or an anoxic/oxic tank, secondary sedimentation tanks, and a sludge thickener. The activated sludge biochemical treatment system process in P-94, P-440, and P-450 adopts an oxic tank **(a)**, while the P-320 system utilizes an anoxic/oxic tank **(b)**. The red line represents the air delivery line, the orange line represents the sludge delivery line, and the blue line represents the sewage pipeline. Black arrows represent wastewater flow direction.

The four PWWTPs differed not only in process configuration but also in influent composition characteristics. Systems P-94 and P-320 primarily treated mixtures of petroleum refinery wastewater and industrial wastewater, whereas Systems P-440 and P-450 functioned as downstream polishing plants receiving different wastewater mixtures, with System P-450 receiving a substantial domestic sewage contribution. The field systems simultaneously varied in process configuration and influent composition. Therefore, comparisons between the O and A/O systems were interpreted as comparisons among full-scale engineering systems rather than as a controlled evaluation of process configuration alone.

The primary treatment processes included oil-water separation, mixing, dissolved air flotation, biological treatment (oxic and anoxic/oxic tank), secondary sedimentation, and sludge thickening (Figure 1). Following biological treatment, the effluents were conveyed to downstream treatment facilities and ultimately discharged after meeting the national discharge standard for petrochemical wastewater (GB 31570–2015).

### 2.2 Sampling and physicochemical analysis

Influent and effluent from each treatment system were collected once daily over a 60-day operational period for physicochemical monitoring. Chemical oxygen demand (COD), ammonia nitrogen (NH_4_^+^-N), total nitrogen (TN), total petroleum hydrocarbons (TPH), and phosphate were determined according to the Chinese standard methods (S.E.P.A., 2002) (Supplementary Table S1). pH was measured using a portable pH meter (Thermo Scientific, United States). Each physicochemical parameter was analyzed in triplicate. Activated sludge for microbial community analysis was collected from the P-320, P-94, P-440, and P-450 systems. Sampling was conducted on Days 30 and 60, representing two relatively stable operational stages during the monitoring period. At each sampling event, six independent activated sludge samples were randomly collected from different locations within each tank to capture spatial heterogeneity. Consequently, the sequencing dataset comprised 60 biological samples (5 sampling locations × 2 sampling times × 6 biological replicates). The samples were immediately transported to the laboratory in an icebox and were stored at −80 °C until DNA extraction. This design supports exploratory association analysis but provides limited power for long-term temporal dynamics, core-community stability, and causal inference.

The removal efficiency was calculated as follows:

$$\eta =\frac{Mi-Me}{Mi}\times 100\%$$

η: the pollutant removal efficiency, %;

*Mi*: the pollutant concentration in influent, mg/L.

*Me*: the pollutant concentration in effluent, mg/L.

### 2.3 DNA extraction and PCR amplification

Total microbial DNA was extracted from activated sludge samples using a SPINeasy™ DNA Pro Kit for Soil (MP Biomedical, Irvine, CA, United States) following the manufacturer’s instructions. A Nanodrop 2000 spectrophotometer (NanoDrop Technologies, Wilmington, DE, United States) was used to measure the DNA concentration and quality. The V4-V5 region of the 16S rRNA gene was amplified using primers 515F (5′-GTGYCAGCMGCCGCGGTAA-3′) and 926R (5′-CCGYCAATTYMTTTRAGTTT-3′) (Parada et al., 2015). All PCR mixtures were supplemented with 15 µL of Phusion® High-Fidelity PCR Master Mix (New England Biolabs), 0.2 µM primers, and 10 ng of genomic DNA template. The PCR protocol involved an initial denaturation at 98 °C for 1 min, followed by 30 cycles of denaturation at 98 °C for 10 s, annealing at 50 °C for 30 s, and extension at 72 °C for 30 s, with a final extension at 72 °C for 5 min. The PCR products were then purified using magnetic beads, mixed in equal amounts based on their concentration, thoroughly mixed, and the target bands were detected and recovered. Furthermore, library construction was performed, and the constructed libraries were quantified using Qubit and Q-PCR. Once the libraries met the quality criteria, they were sequenced on the Illumina Nova6000 platform using PE250 chemistry, acquiring 2 × 250 bp paired-end reads. The original sequence data have been submitted to the China National Bioinformatics Center (https://ngdc.cncb.ac.cn/gsa) - the National Genomic Data Center Genomic Sequence Archive (CNCB-NGDC) (PRJCA038138), with the GSA submission number being CRA024629.

### 2.4 Bioinformatics processing

Raw sequencing data were demultiplexed according to barcode and primer sequences. After barcode and primer trimming, paired-end reads were merged using FLASH (v1.2.11; http://ccb.jhu.edu/software/FLASH/) (Magoč and Salzberg, 2011). Cutadapt was then used to identify and remove residual reverse primer sequences. The merged reads were subjected to quality control, and USEARCH v11 (Edgar et al., 2011) was used for denoising, chimera removal, and generation of zero-radius operational taxonomic units (zOTUs) (Picazo et al., 2019). Taxonomic annotation was conducted using QIIME2 (v2023.2) in the original pipeline (Caporaso et al., 2010). The zOTU abundance table was rarefied to an equal sequencing depth of 27,946 to minimize bias caused by differences in sequencing depth. The resulting zOTU table was used for downstream diversity, differential-abundance, correlation, iCAMP, and PLS-PM analyses.

### 2.5 Statistical analysis

All statistical analyses were performed in R (v4.3.3; http://www.r-project.org/). Alpha-diversity indices (Shannon, Simpson, richness, and Pielou’s evenness) and Bray-Curtis dissimilarities were calculated using the ‘vegan’ package. Principal coordinate analysis (PCoA) based on Bray-Curtis dissimilarity was used to visualize differences among sample groups and was plotted with ‘ggplot2’. Shared and unique zOTUs were summarized using the ‘VennDiagram’ package (v1.7.3) and visualized with ‘ggvenn’.

Microbial biomarker identification was performed using Linear Discriminant Analysis Effect Size (LEfSe) implemented in the ‘microeco’ R package, following a three-stage analytical framework (Liu et al., 2021). Core zOTUs were defined using a conservative threshold: occurrence in 100% of samples within the target comparison group and total relative abundance >0.1% (Chen et al., 2022). This stringent criterion was selected to identify highly persistent taxa under full-scale operating conditions (Supplementary Table S2). Raw zOTU tables were normalized to relative abundance and log10-transformed to mitigate compositional bias.

Taxonomic hierarchy was retained from the phylum to the species level. First, Kruskal–Wallis tests (α = 0.05) identified features with significant abundance differences among groups. Second, pairwise Wilcoxon rank-sum tests were used for subgroup comparisons. Finally, Linear Discriminant Analysis (LDA) quantified effect sizes, and biomarkers with LDA scores >4 or >4.5, depending on the comparison shown in the corresponding figure, were retained after 1,000 bootstrap iterations.

Additional statistical tests, including niche breadth comparisons, Kruskal–Wallis tests, t-tests, and rank-sum tests, were implemented in R. Significance levels are reported as *P < 0.05, **P < 0.01, ***P < 0.001, and ****P < 0.0001 (P values were adjusted using the Benjamini–Hochberg false discovery rate (FDR) procedure).

### 2.6 Inference of community assembly of the bacteria in activated sludge

Microbial community assembly patterns were quantified using the ‘icamp’ package in R, which employs phylogenetic bin-based null model analysis to classify community turnover into statistical fractions associated with homogeneous selection (HoS), heterogeneous selection (HeS), dispersal limitation (DL), homogenizing dispersal (HD), and drift or undominated processes (DR) (Ning et al., 2020). Taxa were partitioned into phylogenetic bins based on evolutionary relationships, and the relative importance of each fraction was estimated using the beta-nearest taxon index (βNTI) and modified Raup-Crick metric (RC).

The key parameters included a minimum bin size threshold (bin.size.limit = 36, optimized via phylogenetic signal testing), 1,000 randomizations for null model construction, and parallel computation (nworker = 4). Environmental variables were integrated to evaluate niche differentiation, while abundance-weighted aggregation quantified process contributions across bins. Statistical significance was determined using |βNTI| > 1.96 and |RC| > 0.95 thresholds. Because null-model outputs are sensitive to sampling structure, environmental heterogeneity, and sequencing depth, terms such as HoS, HeS, DL, HD, and DR are used here as model-based classifications rather than direct observations of ecological mechanisms. Phylogenetic trees were visualized using the iTOL platform (https://itol.embl.de) in circular layout mode, with branch lengths preserved and tip labels aligned at leaf nodes.

All analyses were conducted in R v4.2.2 with code adapted from Ning et al. (2020) and validated against simulated datasets.

### 2.7 PLS-PM construction for COD removal associations

Partial least squares path modeling (PLS-PM) was implemented using the ‘plspm’ package in R to evaluate multivariate associations among measured operational, chemical, and microbial variables (Tenenhaus et al., 2005). To focus on the primary pollutant (COD), subsequent analyses were conducted using COD as the representative response variable. The model integrated all manifest variables, which were mapped to seven latent constructs: process configuration, influent composition, influent nutrient condition (Tre-in), effluent residual pollutant condition (Tre-ef), bacterial community, influent COD, and COD removal rate. The model structure was adjusted according to absolute loading values, and indicators with loadings substantially below 0.7 were evaluated for exclusion. Data standardization (scale = TRUE) was applied to mitigate scale heterogeneity. The inner model defined directional pathways based on prior engineering and ecological assumptions, while the outer model specified reflective relationships between constructs and their indicators using mode A parameterization.

Bootstrap validation (boot.val = TRUE) with 1,000 resamplings assessed path coefficient stability and model adequacy, including goodness-of-fit (Gof) and cross-loadings. Significant pathways were identified at |t-value| > 1.96 (α = 0.05). Finally, path diagrams were generated using the script *innerplot*.

## 3 Results

### 3.1 Pollutant removal performance of the full-scale PWWTPs

The treatment performance of the investigated full-scale PWWTPs was evaluated based on pollutant removal efficiencies and compliance with the emission standard (GB 31571–2015). In the A/O process (P-320), COD removal ranged from 58.6% to 75.9%, while NH_4_^+^-N and TN removal varied within 32.2%–73.5% and 38.0%–64.9%, respectively (Supplementary Table S1). TPH removal ranged from 37.8% to 85.5%, and phosphate removal reached 46.5%–58.3%. In the O processes (P-94, P-440, and P-450), P-450 showed the highest COD (87.8%) and NH_4_^+^-N (85.1%) removal, while P-440 and P-450 achieved the highest TPH removal (91.8% and 92.9%, respectively). The P-94 exhibited the lowest COD removal among the O processes.

Overall, P-450 exhibited the highest removal performance among the O processes, whereas P-94 underperformed in meeting COD and TN limits. The A/O process removed COD and NH_4_^+^-N effectively but showed moderate TN removal. All systems achieved phosphate compliance.

### 3.2 Diversity distribution patterns across influent composd itions and processes in the PWWTPs

In total, 12,746 zOTUs were identified from the 60 samples collected from the four PWWTP systems. Alpha diversity indices (Shannon, Pielou’s evenness, Simpson, and Richness) described bacterial diversity across wastewater types and process configurations (Figure 2). Among the three O processes, richness and diversity were highest in P-450, followed by P-94, whereas P-440 had the lowest species richness and diversity (Figure 2a). Significant differences were observed between P-94 and P-450 (*P* < 0.001). The O processes also showed higher community richness and diversity than the A/O processes (*P* < 0.001) (Figure 2b). This pattern was a system-level field observation and cannot be attributed exclusively to process configuration. More sample experiments need to be designed to confirm this.

**FIGURE 2:**
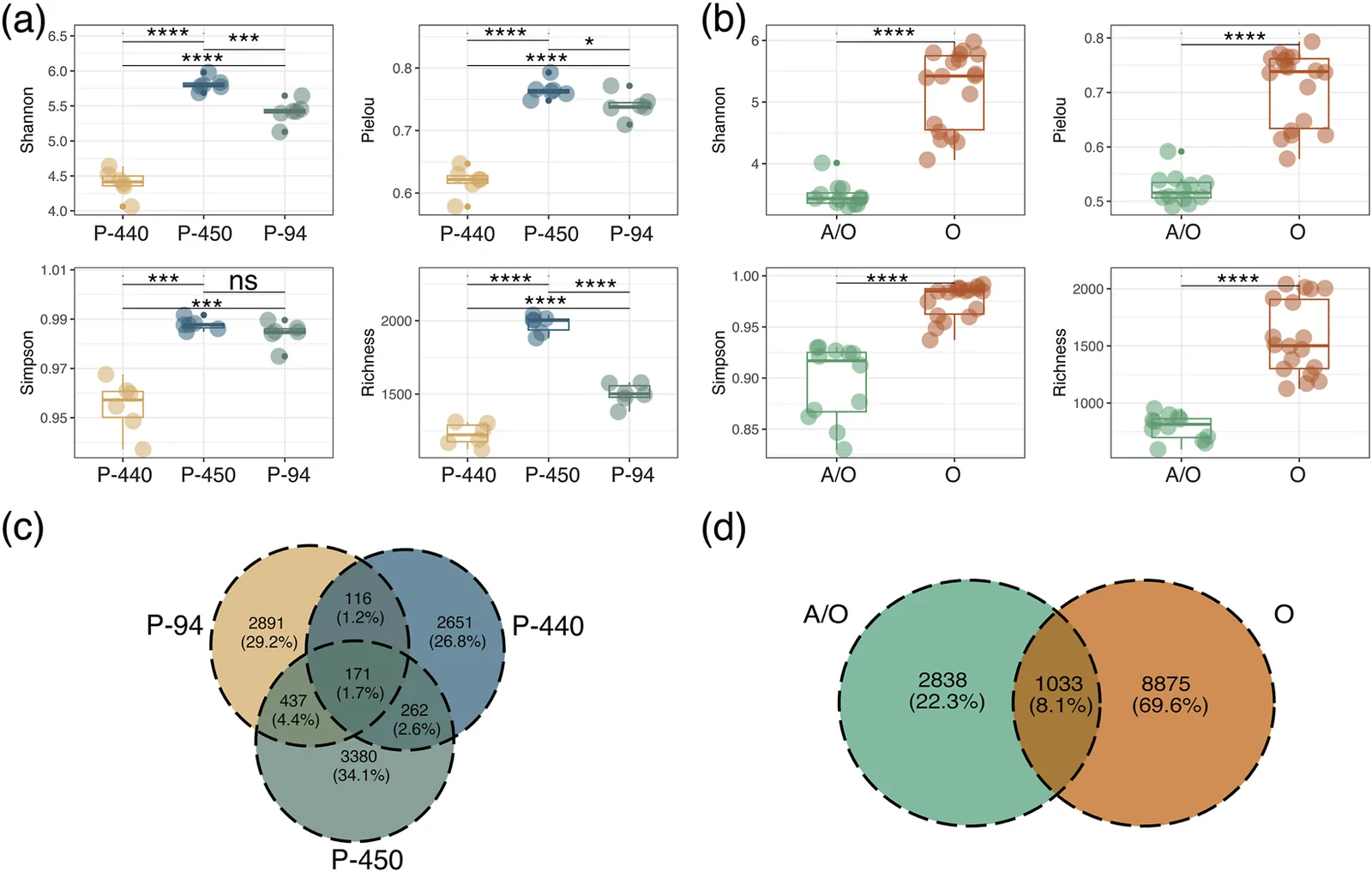
Changes in microbial community diversity and composition across wastewater compositions and process configurations. Alpha diversity indices (Shannon, Pielou’s evenness, Simpson, and richness) describe activated sludge bacterial communities across wastewater compositions (P-94, P-440, and P-450) **(a)** and process configurations (A/O and O) **(b)**. Differences were tested using t-tests and the Kruskal–Wallis tests shown in the figure (ns: *P* > 0.05, *P < 0.05, **P < 0.01, ***P < 0.001, ****P < 0.0001), P values were adjusted using the FDR. Horizontal bars within boxes represent medians; box tops and bottoms represent the 75th and 25th percentiles, respectively. Venn diagrams show the number and percentage of unique and shared zOTUs among influent compositions **(c)** and process configurations **(d)**.

The Venn diagrams (Figures 2c,d) depicted substantial differences in activated sludge microbial community composition among samples. Only 171 core zOTUs (1.7%) were shared by the three O processes (Figure 2c), whereas 8.1% (1033 zOTUs) were shared between A/O and O samples (Figure 2d). This contrasting pattern is noteworthy and suggests that the degree of community overlap differs depending on the level at which the systems are compared. These results indicate strong community differentiation among the sampled systems, likely reflecting combined effects of influent source, process configuration, and local operation. Bray-Curtis distance-based PCoA further showed separation among A/O and O samples (Supplementary Figure S1a), as well as among P-94, P-440, and P-450 (Supplementary Figure S1b).

### 3.3 Distribution pattern of bacterial taxonomic compositions and biomarkers

The top 14 bacterial phyla are shown, with the remaining taxa categorized as “Others”. At the phylum level (Figures 3a,b), Proteobacteria dominated the three O systems, accounting for 43.29%–55.74% in P-440, 51.84%–58.21% in P-450, and 44.27%–72.02% in P-94. Bacteroidota also varied among influents, with relative abundances of 4.85%–14.74% in P-440, 17.18%–22.14% in P-450, and 7.09%–18.59% in P-94. Acidobacteriota were abundant in P-440 (24.65%–36.73%) but less abundant in P-450 (2.54%–3.89%) and P-94 (1.89%–5.69%). In contrast, Planctomycetota were more abundant in P-450 (9.01%–14.27%) and P-94 (3.50%–9.71%) than in P-440 (0.83%–1.69%).

**FIGURE 3:**
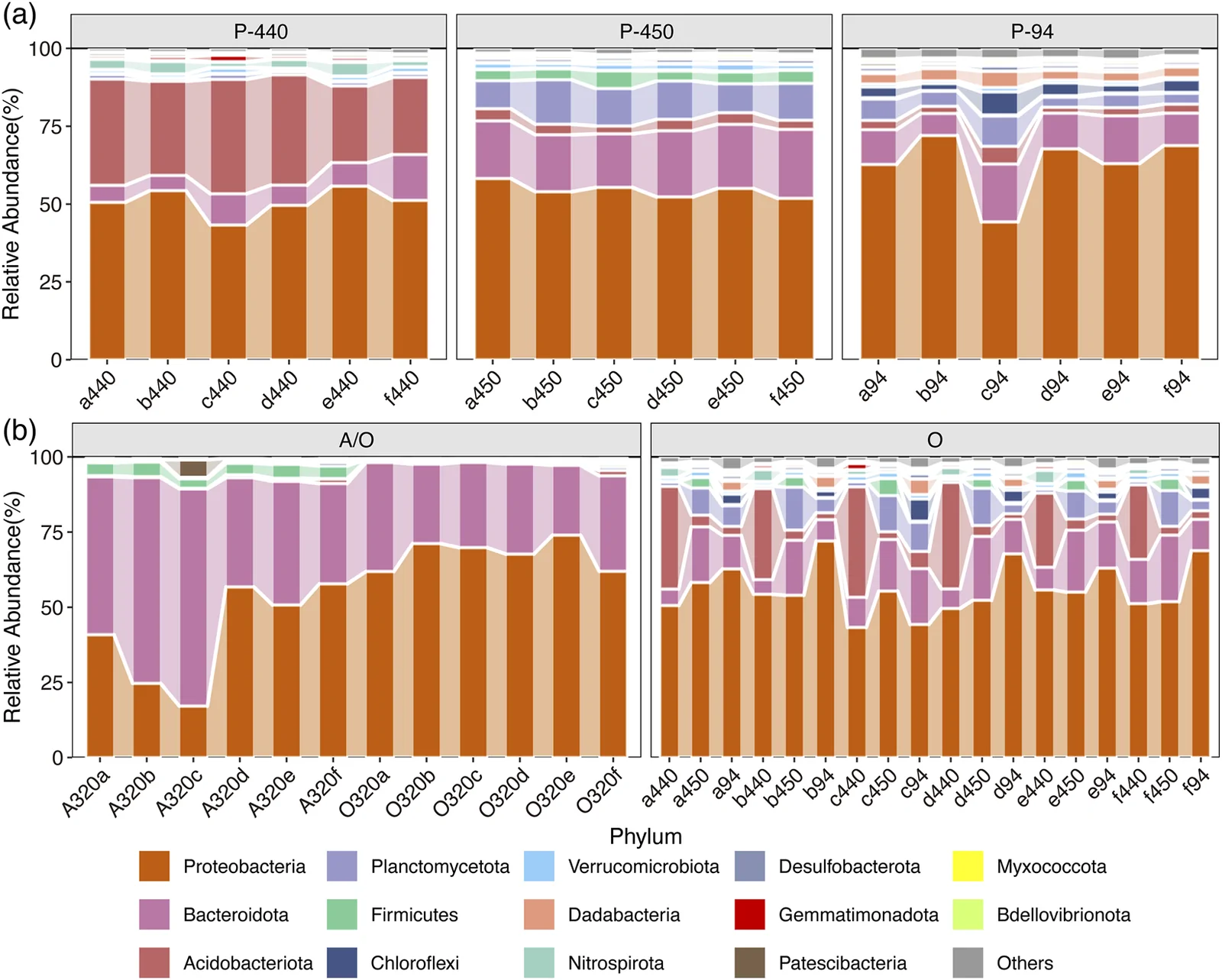
Taxonomic distribution patterns of activated sludge microbial communities. Relative abundance of bacterial communities at the phylum level across wastewater compositions (P-94, P-440, and P-450) **(a)** and process configurations (A/O and O) **(b)**. The top 14 phyla are shown, with the remaining taxa classified as “Others”.

At the class level (Supplementary Figure S2), Gammaproteobacteria were predominant in the three O processes. Bacteroidia and Alphaproteobacteria were also common, although their relative abundances differed among systems. Acidobacteriae were abundant in P-440 (16.23%–30.65%) but scarce in P-450 and P-94 (Supplementary Figure S2a). In the A/O comparison, Proteobacteria and Bacteroidota accounted for more than 90% of the bacterial community (Supplementary Figure S2b), whereas the O systems contained higher proportions of Proteobacteria, Bacteroidota, Acidobacteriota, and Planctomycetota. Figure 4b further showed that Gammaproteobacteria, Bacteroidia, and Alphaproteobacteria dominated the A/O samples (>80%), while Acidobacteriae, Planctomycetes, and Holophagea were enriched in the O samples.

**FIGURE 4:**
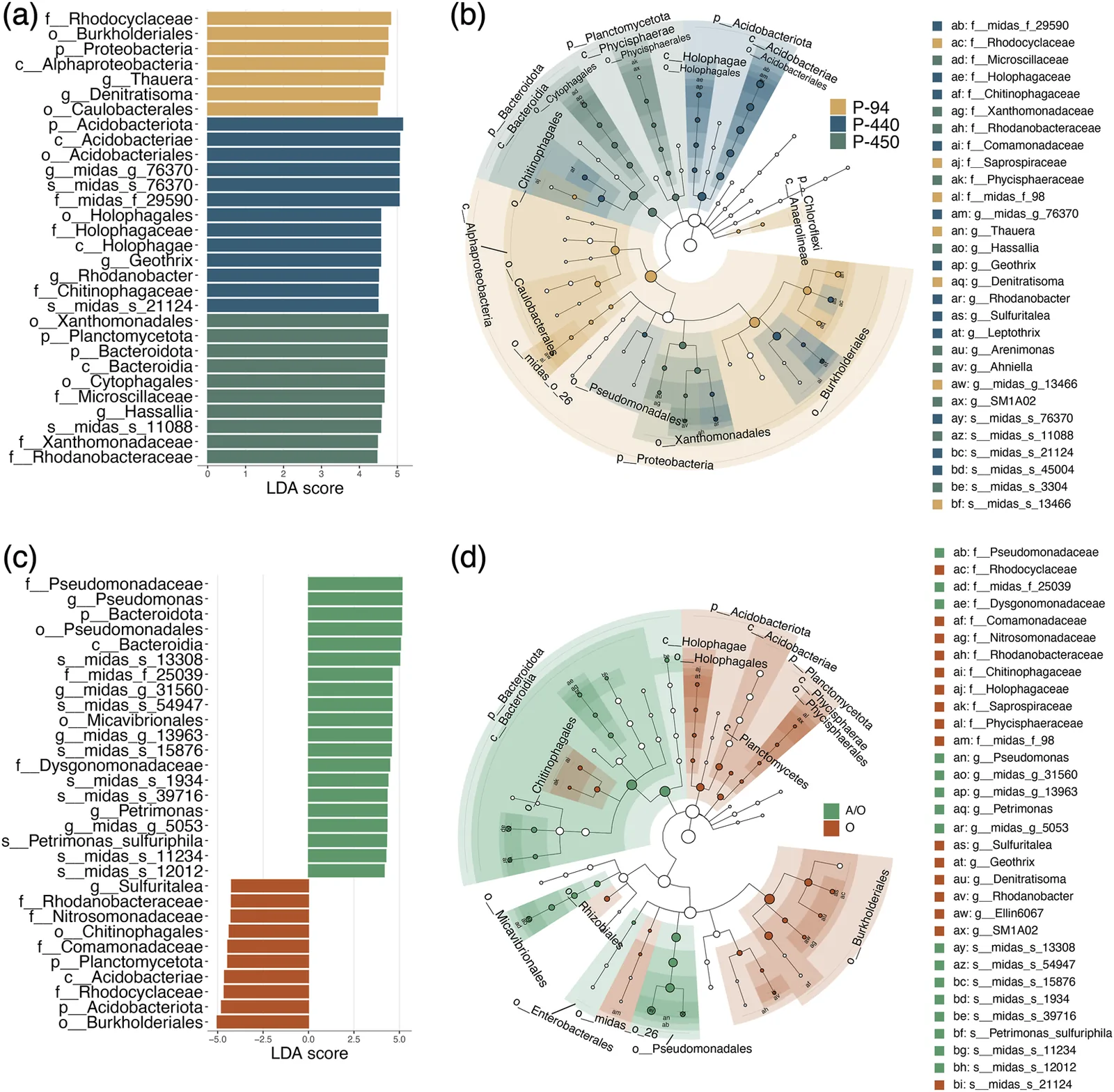
Species distribution patterns in activated sludge across different influent compositions **(a,b)** and different PWWTP treatment processes **(c,d)**. The distribution of LDA values in the histogram shows the species with an LDA score greater than 5 **(a,c)**. Cladogram of LEfSe for bacterial biomarkers enriched in each treatment layer **(b,d)**. The circles radiating from the inside to the outside of the branch diagram represent the classification level from kingdom to species. p, c, o, f, g, and s represent phylum, class, order, family, genus, and species. Each small circle at different classification levels represents a classification at that level, and the diameter of the small circle is proportional to the relative abundance. Colored dots symbolize Taxa with distinct differences between samples. The species names on the right indicate the biomarkers not shown in the figure, with the corresponding letter–number codes matching the figure.

LEfSe analysis revealed differentially abundant bacterial taxa among wastewater types (Supplementary Figure S3a), with taxa retained at LDA scores >4.5. Proteobacteria were the most differentially abundant phylum in P-94, whereas Acidobacteriota and Proteobacteria were enriched in OP-440. Planctomycetota and Proteobacteria were enriched in P-450 (Supplementary Figure S3b). In the A/O system, Bacteroidota and Proteobacteria were differentially abundant in P-320 (Supplementary Figure S3b), indicating community differences between compartments within the A/O process. LEfSe analysis (Figures 4a,b) identified Rhodocyclaceae, Burkholderiales, and Proteobacteria as enriched taxa in P-94; Acidobacteriota, Acidobacteriae, and Acidobacteriales as enriched taxa in P-440; and Xanthomonadales, Planctomycetota, and Bacteroidota as enriched taxa in P-450.

Furthermore, LEfSe analysis was performed to identify taxa that were differentially abundant between the A/O and O systems (Figures 4c,d). Together with alpha and beta diversity analyses, these results indicate that bacterial community composition varied across both influent compositions and process configurations.

### 3.4 Distribution characteristics of core zOTUs and correlations with system performance

We report the richness percentage and relative abundance of core zOTUs versus the remaining bacterial zOTUs in different PWWTP treatment processes. The A/O process contained 36 core zOTUs (4.57% richness), whereas the O processes contained 11 core zOTUs (0.7% richness), accounting for 51.97% and 2.87% of all sequences in A/O and O samples, respectively (Figures 5a,b). The distribution of core zOTUs across samples showed that the overlapping zOTUs were mainly affiliated with Gammaproteobacteria, Alphaproteobacteria, and Bacteroidia (Figure 5c). Spearman’s rank correlation analysis was used to examine associations between pollutant removal rates and core zOTUs in A/O and O processes (Figures 5d,e). Micavibrionales (zOTU2), Rhodobacteraceae (zOTU98), Petrimonas (zOTU8), Cloacibacterium (zOTU32), and Candidatus Berkiella (zOTU679) were positively correlated with COD and TN removal in the A/O process. Rhodocyclaceae (zOTU176) and Hydrogenophaga (zOTU228) were positively correlated with COD removal in the O processes. The classification information of each core zOTU is detailed in Supplementary Table S1.

**FIGURE 5:**
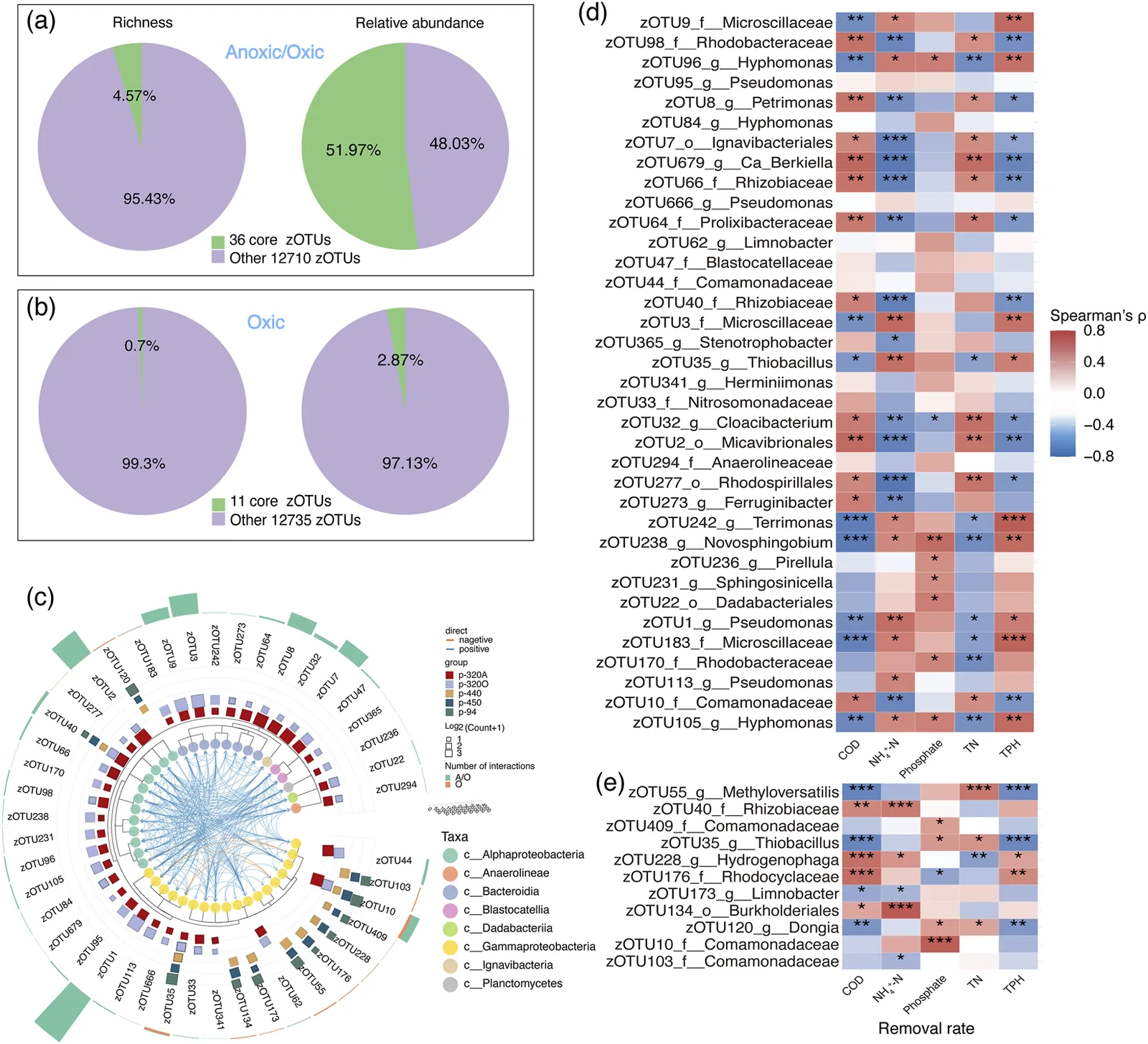
Composition and potential indicator value of core zOTUs. Percentage and relative abundance of core zOTUs and remaining zOTUs in the activated sludge microbial communities of the A/O **(a)** and O systems **(b)**. Distribution of core zOTUs across samples **(c)**. Spearman’s rank correlation analysis shows associations between pollutant removal rates and core zOTUs in the A/O **(d)** and O systems **(e)**. The color gradient indicates Spearman’s rank correlation coefficients, with dark red indicating stronger positive correlations and dark blue indicating stronger negative correlations (*P < 0.05, **P < 0.01, ***P < 0.001).

In the A/O treatment group, activated sludge bacterial community composition was significantly correlated with several pollutant variables (Supplementary Figure S4). The strongest correlations were observed for influent TN (r = 0.646), effluent TPH (r = 0.619), and influent COD (r = 0.480). In the O group, ammonia nitrogen-related variables were prominent: influent NH_4_^+^-N (r = 0. P-440) and effluent NH_4_^+^-N (r = 0.570) were significantly correlated with microbial community composition. These patterns are consistent with potential links between nitrogen transformation and community structure, but functional gene quantification would be required to validate specific metabolic contributions.

### 3.5 Ecological process patterns of activated sludge microbial communities

The iCAMP was employed to investigate the effects of different influent compositions and process configurations on the relative importance of deterministic and stochastic community assembly (Figures 6a,b). Between the process configurations, deterministic fractions accounted for 27.52% of the total number of bins and 32.29% of the relative abundance, whereas stochastic fractions accounted for 72.48% of the bins and 67.71% of the relative abundance (Figure 6a). Proteobacteria dominated many bins, accounting for more than 50% of total relative abundance, with dominant genera including *Pseudomonas*, *Rhodanobacter*, *Leptothrix*, and *Denitratisoma*, *etc.* Pearson’s heat map showed that bin57 (Bacteroidota) was highly positively correlated with COD removal (r = 0.51) and NH_4_^+^-N removal (r = 0.48). HoS and HeS fractions were higher in the A/O process (50.1%) than in the O processes (23.6%), whereas HoS (49.8%) and DL (50.2%) were the dominant classifications in the A/O and O comparisons, respectively. The O systems showed wider niche breadth than the A/O system (Figure 6c). These patterns suggest stronger environmental filtering signals in the A/O samples and stronger stochastic or dispersal-related signals in the O samples. Still, they should not be interpreted as definitive evidence of process-driven mechanisms because influent and operational variables were confounded.

**FIGURE 6:**
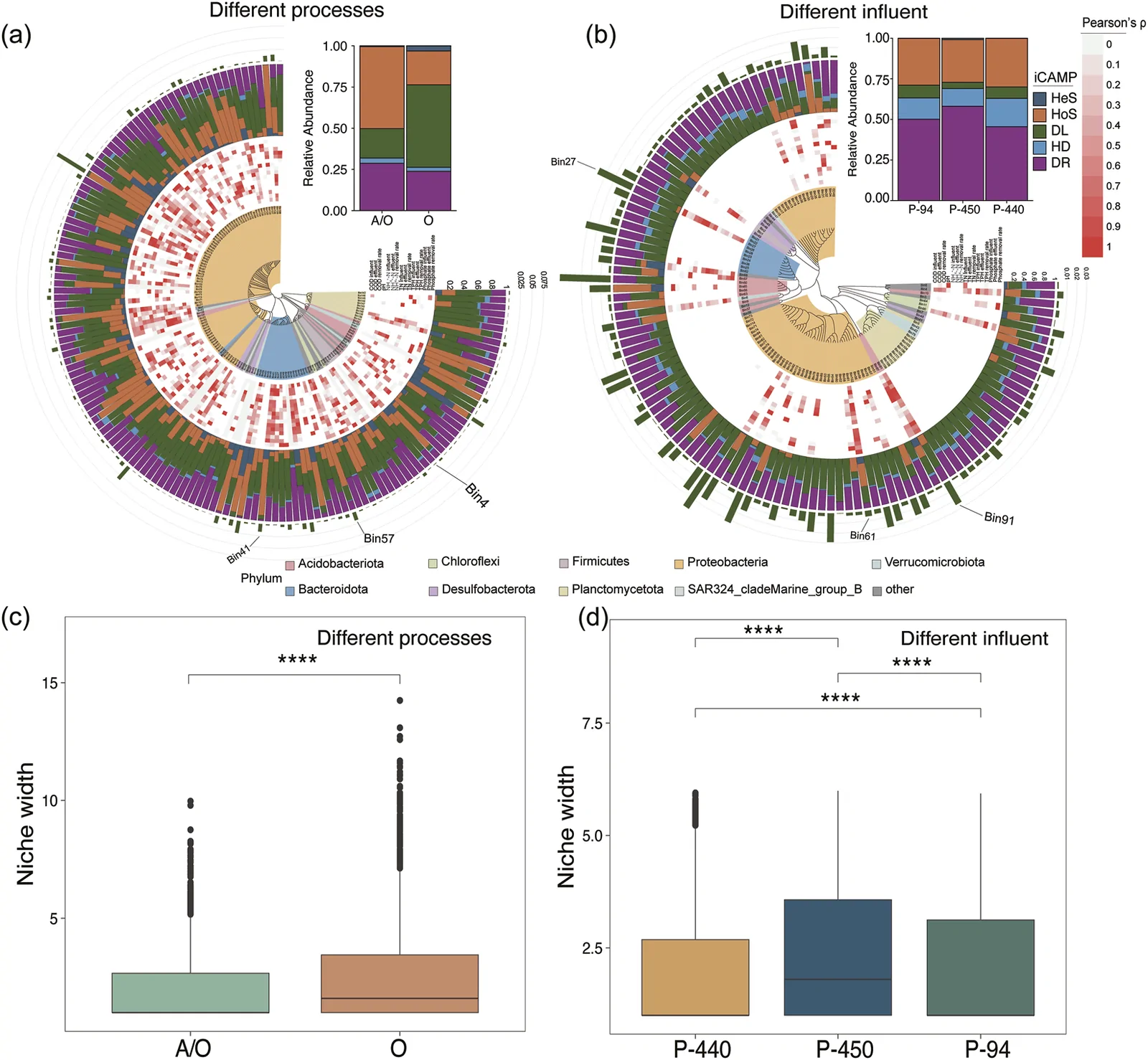
Importance of ecological processes of the activated sludge microbial communities. **(a)** Process configurations (A/O and O); **(b)** influent compositions (P-94, P-440, and P-450). Branches and inner strips are colored by phylum (first annulus). The heatmap shows relationships between each bin and wastewater properties, including influent and effluent COD, pH, NH_4_^+^-N, TN, TPH, phosphate, and corresponding removal rates (second annulus). Stacked bars represent the relative abundance of different ecological process classifications (third annulus). Green bars represent the relative abundance of each bin (fourth annulus). Boxplots show mean habitat niche breadth for all taxa across process configurations **(c)** and influent compositions **(d)**. ****P < 0.0001.

Between influent compositions, deterministic fractions accounted for 27.52% of total bins and 32.29% of relative abundance, whereas stochastic fractions accounted for 72.48% of bins and 67.71% of relative abundance (Figure 6b). Proteobacteria and Bacteroidota were the dominant phyla in the phylogenetic branches, accounting for more than 50% and 20%–25% of total relative abundance, respectively. The bin-level heat map indicated that bin61 was positively correlated with several indicators, especially COD (r = 0.32) and NH_4_^+^-N (r = 0.59) removal efficiencies. The DR fraction was highest in P-450 (58.1%), followed by P-94 (50.1%) and P-440 (45.5%) (Figure 6b). P-450 also showed wider niche breadth (Figure 6d), which may be related to its domestic sewage contribution and lower selective pressure.

### 3.6 Relationships among process configuration, influent composition, bacterial community, and COD removal

PLS-PM was used to explore multivariate associations among process configuration, influent composition, influent COD, Tre-in, Tre-ef, bacterial community, and COD removal (Figure 7). As shown in Figure 7a, the GoF for model is 0.771, process configuration was positively associated with bacterial community (β = 0.966, *P* < 0.001). Tre-in explained a large proportion of the variance in Tre-ef (*R* ^2^ = 0.928, β = 0.642, *P* < 0.001). Process configuration was not directly associated with COD removal (β = −0.185). Path effect decomposition further indicated indirect relationships among variables (Figure 7c). Because the model was fitted to a limited observational dataset, these pathways should be interpreted as association patterns rather than causal proof.

**FIGURE 7:**
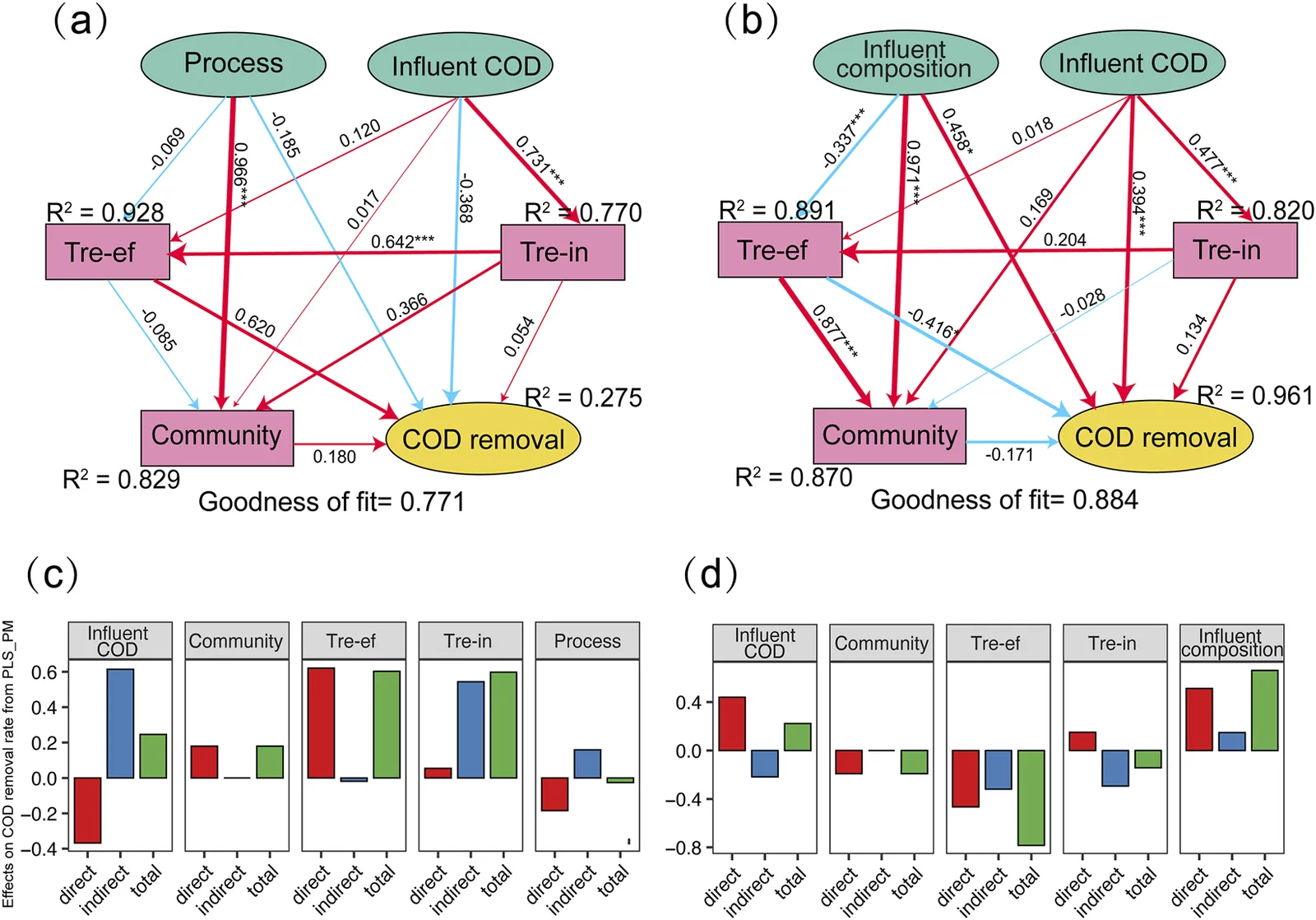
Relationships among process configuration, influent composition, influent COD, influent and effluent pollutant conditions (Tre-in and Tre-ef), bacterial community, and COD removal rate. **(a)** The partial least squares path model (PLS-PM) illustrates the relationships among the PWWTP process, influent COD, Tre-in, Tre-ef, bacterial community, and COD removal. **(b)** PLS-PM reveals the relationships among influent compositions, influent COD, Tre-in, Tre-ef, bacterial community, and COD removal. Line thickness is proportional to path coefficient values; red arrows represent positive associations and blue arrows represent negative associations (*P ≤ 0.05, **P ≤ 0.01, ***P ≤ 0.001). Standard path coefficients are shown near pathway arrows. *R* ^2^ denotes the proportion explained by independent latent variables. Bar graphs show the direct, indirect, and total effects of influent COD, bacterial community structure, Tre-ef, Tre-in, and process configuration **(c)**, and influent composition **(d)**, on COD removal rate. All data were log-transformed.

Directed graph showing the difference in the total effects on responding to influent composition between influent COD, Tre-in, Tre-ef, and bacterial communities. The PLS-PM model showed acceptable exploratory fit (GoF = 0.884). Influent composition was positively associated with bacterial community structure (β = 0.971, *P* < 0.001) and negatively associated with Tre-ef (*R* ^2^ = 0.891, β = −0.337, *P* < 0.001) (Figure 7b). Influent composition and influent COD were directly associated with COD removal, whereas bacterial community structure and Tre-ef showed mainly indirect associations (Figure 7d). These results support the interpretation that influent characteristics were more closely linked to COD removal than process category in the current dataset.

## 4 Discussion

PWWTPs treat toxic and recalcitrant organic-polluted water and are therefore important systems in environmental engineering (Alabi, 2022). However, knowledge remains limited regarding activated sludge bacterial communities in full-scale petrochemical biochemical treatment systems and their relationships with treatment efficiency (Chen et al., 2022; Guo et al., 2017; Kim et al., 2020). The present study provides field-scale evidence that microbial diversity, community composition, community assembly, and COD removal covary with influent composition and process configuration. Because the dataset is observational, focusing on plausible interpretations and engineering implications.

### 4.1 Community structure and potential bioindicators in PWWTPs

Studying full-scale systems is crucial for ensuring that findings are applicable to real-world operation (Chen et al., 2022; Kim et al., 2020). In this study, community diversity was higher in the O system than in the A/O system (Figure 2a). This pattern is consistent with previous studies showing that aerobic environments often support greater bacterial diversity than oxygen-limited habitats (Wang Y. et al., 2024; Zha et al., 2021), although the present field systems also differed in influent characteristics and operational conditions.

Acidobacteriota and Planctomycetota were abundant in the O processes (Figure 3a) and are widely found in wastewater treatment environments. Members of these phyla have been associated with organic matter transformation and nutrient cycling (Kristensen et al., 2021; Zhang et al., 2023). The high proportion of unique zOTUs in O processes (Figure 2d) indicates strong community differentiation among sites. The enrichment of Bacteroidota and Pseudomonadaceae in the A/O process may be consistent with adaptation to alternating redox conditions and organic matter transformation (Hu et al., 2023; Li et al., 2023), but this inference requires functional validation. Meanwhile, compared to samples from P-450 (main domestic wastewater), P-94 samples (high-concentration COD and high oily wastewater) showed significantly lower bacterial community diversity (Figure 2a), indicating that high pollutant load was associated with reduced community diversity by inhibiting the growth of some sensitive taxa. This result was consistent with previous research in coking wastewater, evincing concentrated polycyclic aromatic hydrocarbons (PAHs) that inhibit microbial activity by inducing oxidative stress (Cao et al., 2023).

Proteobacteria were abundant across systems and include many taxa with broad substrate utilization potential (Cui et al., 2024). The enrichment of *Thauera* in P-94, which received high-COD and oily wastewater, is consistent with previous reports that some *Thauera* species can participate in aromatic compound degradation and nutrient removal (Ren et al., 2021). Nevertheless, taxonomic enrichment alone cannot confirm *in situ* metabolic activity. Several core zOTUs belonged to genera or families previously linked to pollutant transformation. For example, *Pseudomonas* showed a significantly positive correlation with COD removal, and *Thiobacillus* was positively correlated with TN removal. *Pseudomonas* has various ecological and biotechnological applications, including promoting plant growth and biocontrol capabilities in cold environments (Chauhan et al., 2023). This bacterium showed excellent pollutant removal performance in constructed wetlands (Wang et al., 2025). These correlations suggest potential bioindicators for monitoring, but they are not yet direct evidence that these taxa drove pollutant removal in the sampled systems.

Accordingly, the practical value of the core zOTU analysis lies mainly in ecological diagnosis. For example, changes in *Candidatus Berkiella*, Rhodobacteraceae, Rhodocyclaceae, *Pseudomonas*, or *Thiobacillus* may help flag shifts in community state under different influent and redox conditions.

### 4.2 Process configurations drive bacterial community assembly in PWWTPs

Various ecological mechanisms may affect the composition and function of microbial communities, reflected in the microbial assembly process (stochastic and deterministic) (Zhou and Ning, 2017). Bacteroidota and Pseudomonadaceae predominated in the A/O process. These microbiotas are adaptable to complex substrate degradation environments and efficiently decompose organic matter under anaerobic conditions (Wang S. et al., 2021). Due to the strong environmental selection effect of the A/O process during operation, the microbial community diversity was less diverse but showed higher stability and functional specificity. This phenomenon has also been reported in the study of municipal sewage treatment plants, indicating that the A/O process has a strong ecological screening effect (Wang et al., 2022).

Unlike A/O, the community assembly mechanism of the O process is mainly driven by the DL, which is consistent with the study that investigated microbial community assembly from large-scale sampling of aeration tanks throughout China (Sun et al., 2020). The reason may be that the O process’s sole aerobic nutrient is conducive to colonizing microorganisms, thus promoting increased community diversity (Flynn et al., 2017). In the present study, Acidobacteriota, Planctomycetota, and Verrucomicrobiota were significantly enriched in the O process assembly but were almost absent in the A/O process. These microbiotas are adaptable to their environment, thriving in high-oxygen conditions and contributing to organic matter degradation and nitrification processes (Flieder et al., 2021; Schmitz et al., 2021). Interestingly, a larger number of zOTUs were shared between the A/O and O processes than among the three individual oxic processes. This pattern does not necessarily indicate that the two process configurations supported more similar bacterial communities. Instead, it likely reflects differences in the process configurations (Cheng et al., 2024).

Among the three oxic processes, each receiving distinct influent compositions and operating under site-specific conditions, thereby imposing a more stringent criterion for defining shared taxa. The wastewater treatment plants employing similar treatment processes may develop plant-specific microbial signatures because influent characteristics and local operational conditions strongly influence community assembly (Yu et al., 2021). The total contribution of the stochastic process was prevalent in different influent compositions, and the DR contribution predominated, whereas the community assembly of domestic sewage was more stochastic (Figure 6b). The bacterial community assembly in the domestic sewage system was stochastic; it might be shaped by weak selection pressure and a wide-species-source ‘relaxed’ niche. The niche width results showed that the oxic process’s microbial community had stronger environmental adaptability. Previous studies also demonstrated that microflora with larger niche widths tend to have more substantial metabolic diversity and can maintain population dominance under variable environmental conditions (Bajić et al., 2021). The limited overlap among the individual oxic process in the present study is more likely attributable to differences in influent composition and plant-specific operation than to aerobic process configuration itself. Therefore, the oxic process was more flexible in adjusting to the microbial community, adapting to the fluctuation of the influent pollutant load, and improving the system’s operational stability.

From an engineering perspective, these ecological indicators may still be informative. A community with broader niche breadth and higher stochastic fractions may be more flexible under moderate influent fluctuation, whereas a community with stronger selection signals may be more specialized and potentially more sensitive to abrupt loading or toxicity shocks. Monitoring shifts in diversity, niche breadth, and assembly classifications complements conventional physicochemical monitoring, especially for early warning of community destabilization.

### 4.3 Factors associated with COD removal and engineering implications

Understanding factors associated with COD removal is crucial for optimizing PWWTP operation. The PLS-PM results suggested that process configuration was not directly associated with COD removal in the current dataset, whereas influent composition and influent COD showed stronger direct associations (Figure 7). This pattern is consistent with the engineering expectation that influent concentration, biodegradability, and toxicity can constrain removal performance even when the same biological treatment principle is applied (Wang Y. et al., 2024). On the contrary, the composition and concentration of influent pollutants, especially COD, TN, and TPH loads, may have shaped the characteristics of influent and effluent pollutants, exerting selective pressure on the activated sludge microbial community, thereby influencing the final treatment efficiency.

The stronger association between influent composition and COD removal suggests several practical implications. First, influent equalization and source control may be as important as biological process adjustment when treating variable petrochemical wastewater. Second, high-COD or oily streams may require pretreatment, staged feeding, or buffering to reduce toxicity and loading shocks before biological treatment (Wang X. et al., 2024). Therefore, controlling the influent concentration is crucial to improving the effluent standard or setting up more hierarchical treatments. Third, microbial indicators should be interpreted together with COD, TN, TPH, phosphate, pH, dissolved oxygen, hydraulic retention time (HRT), sludge age, and reflux strategy, because operational changes that improve removal efficiency may also alter community stability and resilience. However, this study did not consider the regulatory role of temperature and HRT, which may overestimate the effect of influent composition. Temperature and HRT can significantly affect system performance, microbial community dynamics, and sludge characteristics (Meynet et al., 2024; Niu et al., 2023). Future studies may couple dynamic models (such as ASM3) with PLS-PM to improve prediction accuracy (Li et al., 2024).

For engineering management, the results support a diagnostic rather than prescriptive use of microbial ecology. Community diversity, potential bioindicators, and iCAMP classifications may help identify whether performance deterioration is accompanied by community simplification, loss of persistent taxa, or shifts toward stronger selection/stochasticity. Such information could guide targeted checks of influent toxicity, aeration, reflux, and nutrient balance. Future optimization should combine microbial monitoring with controlled operational trials and dynamic process models, such as activated sludge models, to evaluate whether microbial indicators improve process stability, chemical consumption, energy use, and overall engineering economics. To convert these associations into operational targets, future work should combine 16S rRNA profiles with functional gene quantification, metagenomics or metatranscriptomics, enzyme activity assays, and batch tests under controlled COD, nitrogen, TPH, dissolved oxygen, and carbon-to-nitrogen conditions.

## 5 Conclusion

This study provides valuable baseline information regarding activated sludge microbial community structure, inferred community assembly, and COD removal associations under different process configurations and influent compositions in full-scale PWWTPs. The sampled O systems showed higher community richness and broader niche breadth than the A/O system, while community composition differed strongly among influent compositions. Furthermore, iCAMP and PLS-PM analyses suggested that deterministic selection dominated in the A/O system, and that influent characteristics appeared more directly linked to COD removal than process configuration. While core zOTUs were statistically associated with pollutant removal and show promise as prospective ecological indicators, their implications for microbial functional adaptation remain exploratory and require further direct validation. Overall, these findings highlight the potential applications of integrating influent control, process operation, and microbial monitoring for PWWTP optimization. To build upon this foundational dataset and confirm causal mechanisms, future studies should employ paired designs, larger sample sizes, and multi-omics or experimental validation.

## Data Availability

The original sequence data in this study can be accessed from the National Genomics Data Centre at the China National Centre for Bioinformatics (CNCB-NGDC) via the accession numbers PRJCA038138 and GSA accession number CRA024629 (https://ngdc.cncb.ac.cn/gsa).
