# Supplementary material for: Ecological assembly of activated sludge microbiomes under contrasting process configurations and influent regimes
Source: Front Bioeng Biotechnol. 2026 Sep 21;14:1912780. doi: 10.3389/fbioe.2026.1912780 (PMC13634615; doi:10.3389/fbioe.2026.1912780)
Supplement: Supplementary file 1 [file Supplementaryfile1.docx]

SUPPLEMENTARY MATERIAL OF THE ARTICLE:

**Ecological Assembly of Activated Sludge Microbiomes Under Contrasting Process Configurations and Influent Regimes**

**Table S1** Summary of influent and effluent physicochemical characteristics and pollutant removal performance during the 60-day monitoring period in the full-scale petrochemical wastewater treatment plants (PWWTPs)

| PWWTPs  Process | Sampling location | Parameter | Influent range  (mg/L) | Mean influent  (mg/L) | Effluent range  (mg/L) | Mean effluent  (mg/L) | Removal efficiency range  (%) | Mean removal efficiency (%) | Emission standard GB 31571-2015*  (mg/L) |
| --- | --- | --- | --- | --- | --- | --- | --- | --- | --- |
| P-320  (A/O) | Anoxic tank | COD | 1721.0-2637.0 | 2083.0 | 595.0-974.0 | 822.0 | 48.0-66.8 | 58.6 |  |
|  |  | NH_4_^＋^-N | 13.5-18.9 | 16.8 | 10.1-13.9 | 11.3 | 18.6-42.5 | 32.2 |  |
|  |  | TN | 424.0-468.0 | 466.0 | 111.0-232.0 | 164.0 | 56.5-76.3 | 64.9 |  |
|  |  | TPH | 14.3-18.3 | 16.8 | 9.9-11.1 | 10.4 | 30.8-42.6 | 37.8 |  |
|  |  | Phosphate | 0.6-1.2 | 0.8 | 0.2-0.7 | 0.4 | 29.2-63.0 | 46.5 |  |
|  |  | pH | 7.8-8.2 | 7.9 | 7.7-8.2 | 7.9 | ̶ | ̶ |  |
| P-320  (A/O) | Oxic tank | COD | 384.0-1000.0 | 800.5 | 66.0-421.0 | 187.2 | 25.1-93.9 | 75.9 |  |
|  |  | NH_4_^＋^-N | 5.0-28.0 | 11.7 | 1.0-7.0 | 2.7 | 12.5-96.4 | 73.5 |  |
|  |  | TN | 1.0-300.0 | 166.7 | 25.0-236.0 | 98.4 | 2.2-81.6 | 38.0 |  |
|  |  | TPH | 5.2-18.1 | 10.5 | 0.2-7.8 | 1.5 | 53.3-98.5 | 85.5 |  |
|  |  | Phosphate | 0.3-1.3 | 0.6 | 0.1-0.4 | 0.2 | 0.0-88.9 | 58.3 |  |
|  |  | pH | 7.7-8.2 | 7.9 | 7.1-8.6 | 7.7 | ̶ | ̶ |  |
| P-94  (O) | Oxic tank | COD | 421.0-2012.0 | 1236.7 | 261.0-729.0 | 448.8 | 3.2-88.2 | 51.5 |  |
|  |  | NH4^＋^-N | 3.0-10.0 | 4.6 | 0.4-4.0 | 1.7 | 0-90.0 | 61.9 |  |
|  |  | TN | 34.0-471.0 | 147.8 | 16.0-84.0 | 48.4 | 4.8-95.9 | 54.2 |  |
|  |  | TPH | 1.7-19.8 | 9.0 | 0.61-15.4 | 4.9 | 1.7-87.4 | 44.7 |  |
|  |  | Phosphate | 0.1-0.8 | 0.4 | 0.0-0.1 | 0.1 | 0.0-96.0 | 71.9 |  |
|  |  | pH | 7.4-7.8 | 7.6 | 7.2-8.0 | 7.6 | ̶ | ̶ |  |
| P-440  (O) | Oxic tank | COD | 124.0-381.0 | 249.2 | 42.0-74.0 | 56.3 | 53.2-88.2 | 75.5 |  |
|  |  | NH4^＋^-N | 13.0-39.0 | 21.5 | 5.0-33.0 | 15.6 | 5.0-70.0 | 28.7 |  |
|  |  | TN | 12.0-36.0 | 25.9 | 4.0-21.0 | 13.6 | 6.3-78.9 | 44.9 |  |
|  |  | TPH | 6.2-19.4 | 14.5 | 0.3-2.9 | 1.1 | 79.3-98.2 | 91.8 |  |
|  |  | Phosphate | 0.1-0.5 | 0.2 | 0.0-0.2 | 0.1 | 0.0-70.0 | 28.2 |  |
|  |  | pH | 7.5-7.6 | 7.5 | 7.4-7.5 | 7.5 | ̶ | ̶ |  |
| P-450  (O) | Oxic tank | COD | 209.0-571.0 | 380.5 | 31.0-69.0 | 44.0 | 77.1-93.2 | 87.8 | ≤ 60 |
|  |  | NH4^＋^-N | 0.5-13.0 | 5.1 | 0.1-5.0 | 0.6 | 40.0-98.0 | 85.1 | ≤ 8.0 |
|  |  | TN | 5.0-26.0 | 14.4 | 4.0-15.0 | 9.5 | 0.0-72.7 | 32.3 | ≤ 40 |
|  |  | TPH | 4.7-20.0 | 13.9 | 0.3-1.9 | 0.9 | 82.7-98.5 | 92.9 | ≤ 5.0 |
|  |  | Phosphate | 0.1-3.0 | 0.4 | 0.1-0.6 | 0.2 | 0.0-93.3 | 47.2 | ≤ 1.0 |
|  |  | pH | 7.7-7.8 | 7.8 | 7.7-7.8 | 7.8 | ̶ | ̶ | 6-9 |

*Emission standard of pollutants for petroleum refining industry in China.

**Table S2** Taxonomic classification of core zOTUs identified in activated sludge microbial communities from full-scale petrochemical wastewater treatment plants

| id | Kingdom | Phylum | Class | | Order | | Family | | Genus |
| --- | --- | --- | --- | --- | --- | --- | --- | --- | --- |
| zOTU47 | Bacteria | Acidobacteriota | Blastocatellia | | Blastocatellales | | Blastocatellaceae | | - |
| zOTU365 | Bacteria | Acidobacteriota | Blastocatellia | | Blastocatellales | | Blastocatellaceae | | Stenotrophobacter |
| zOTU294 | Bacteria | Chloroflexi | Anaerolineae | | Anaerolineales | | Anaerolineaceae | | midas_g_156 |
| zOTU120 | Bacteria | Proteobacteria | Alphaproteobacteria | | Dongiales | | Dongiaceae | | Dongia |
| zOTU2 | Bacteria | Proteobacteria | Alphaproteobacteria | | Micavibrionales | | midas_f_25039 | | midas_g_31560 |
| zOTU64 | Bacteria | Bacteroidota | Bacteroidia | | Bacteroidales | | Prolixibacteraceae | | midas_g_25828 |
| zOTU32 | Bacteria | Bacteroidota | Bacteroidia | | Flavobacteriales | | Weeksellaceae | | Cloacibacterium |
| zOTU273 | Bacteria | Bacteroidota | Bacteroidia | | Chitinophagales | | Chitinophagaceae | | Ferruginibacter |
| zOTU242 | Bacteria | Bacteroidota | Bacteroidia | | Chitinophagales | | Chitinophagaceae | | Terrimonas |
| zOTU8 | Bacteria | Bacteroidota | Bacteroidia | | Bacteroidales | | Dysgonomonadaceae | | Petrimonas |
| zOTU238 | Bacteria | Proteobacteria | Alphaproteobacteria | | Sphingomonadales | | Sphingomonadaceae | | Novosphingobium |
| zOTU231 | Bacteria | Proteobacteria | Alphaproteobacteria | | Sphingomonadales | | Sphingomonadaceae | | Sphingosinicella |
| zOTU98 | Bacteria | Proteobacteria | Alphaproteobacteria | | Rhodobacterales | | Rhodobacteraceae | | - |
| zOTU84 | Bacteria | Proteobacteria | Alphaproteobacteria | | Caulobacterales | | Hyphomonadaceae | | Hyphomonas |
| zOTU96 | Bacteria | Proteobacteria | Alphaproteobacteria | | Caulobacterales | | Hyphomonadaceae | | Hyphomonas |
| zOTU105 | Bacteria | Proteobacteria | Alphaproteobacteria | | Caulobacterales | | Hyphomonadaceae | | Hyphomonas |
| zOTU170 | Bacteria | Proteobacteria | Alphaproteobacteria | | Rhodobacterales | | Rhodobacteraceae | | - |
| zOTU183 | Bacteria | Bacteroidota | Bacteroidia | | Cytophagales | | Microscillaceae | | midas_g_9811 |
| zOTU3 | Bacteria | Bacteroidota | Bacteroidia | | Cytophagales | | Microscillaceae | | midas_g_13963 |
| zOTU9 | Bacteria | Bacteroidota | Bacteroidia | | Cytophagales | | Microscillaceae | | midas_g_5053 |
| zOTU7 | Bacteria | Bacteroidota | Ignavibacteria | | Ignavibacteriales | | PHOS-HE36 | | midas_g_179 |
| zOTU679 | Bacteria | Proteobacteria | Gammaproteobacteria | | | midas_o_147 | midas_f_2362 | | Ca_Berkiella |
| zOTU277 | Bacteria | Proteobacteria | Alphaproteobacteria | | Rhodospirillales | | midas_f_17292a | | midas_g_17894 |
| zOTU1 | Bacteria | Proteobacteria | Gammaproteobacteria | | | Pseudomonadales | | Pseudomonadaceae | Pseudomonas |
| zOTU113 | Bacteria | Proteobacteria | Gammaproteobacteria | | | Pseudomonadales | | Pseudomonadaceae | Pseudomonas |
| zOTU95 | Bacteria | Proteobacteria | Gammaproteobacteria | | | Pseudomonadales | | Pseudomonadaceae | Pseudomonas |
| zOTU666 | Bacteria | Proteobacteria | Gammaproteobacteria | | | Pseudomonadales | | Pseudomonadaceae | Pseudomonas |
| zOTU134 | Bacteria | Proteobacteria | Gammaproteobacteria | | | Burkholderiales | SC-I-84 | | midas_g_41102 |
| zOTU228 | Bacteria | Proteobacteria | Gammaproteobacteria | | | Burkholderiales | Comamonadaceae | | Hydrogenophaga |
| zOTU44 | Bacteria | Proteobacteria | Gammaproteobacteria | | | Burkholderiales | Comamonadaceae | |  |
| zOTU409 | Bacteria | Proteobacteria | Gammaproteobacteria | | | Burkholderiales | Comamonadaceae | | midas_g_887 |
| zOTU55 | Bacteria | Proteobacteria | Gammaproteobacteria | | | Burkholderiales | Rhodocyclaceae | | Methyloversatilis |
| zOTU22 | Bacteria | Dadabacteria | Dadabacteriia | | | Dadabacteriales | midas_f_603 | | midas_g_603 |
| zOTU176 | Bacteria | Proteobacteria | Gammaproteobacteria | | | Burkholderiales | Rhodocyclaceae | | - |
| zOTU35 | Bacteria | Proteobacteria | Gammaproteobacteria | | | Burkholderiales | Hydrogenophilaceae | | Thiobacillus |
| zOTU173 | Bacteria | Proteobacteria | Gammaproteobacteria | | | Burkholderiales | Burkholderiaceae | | Limnobacter |
| zOTU62 | Bacteria | Proteobacteria | Gammaproteobacteria | | | Burkholderiales | Burkholderiaceae | | Limnobacter |
| zOTU341 | Bacteria | Proteobacteria | Gammaproteobacteria | | | Burkholderiales | Oxalobacteraceae | | Herminiimonas |
| zOTU33 | Bacteria | Proteobacteria | Gammaproteobacteria | | | Burkholderiales | Nitrosomonadaceae | | Ellin6067 |
| zOTU236 | Bacteria | Planctomycetota | | Planctomycetes | Pirellulales | | Pirellulaceae | | Pirellula |
| zOTU103 | Bacteria | Proteobacteria | Gammaproteobacteria | | | Burkholderiales | Comamonadaceae | | - |
| zOTU10 | Bacteria | Proteobacteria | Gammaproteobacteria | | | Burkholderiales | Comamonadaceae | | - |
| zOTU40 | Bacteria | Proteobacteria | Alphaproteobacteria | | | Rhizobiales | Rhizobiaceae | | Allorhizobium-Neorhizobium-Pararhizobium-Rhizobium |
| zOTU66 | Bacteria | Proteobacteria | Alphaproteobacteria | | | Rhizobiales | Rhizobiaceae | | Allorhizobium-Neorhizobium-Pararhizobium-Rhizobium |

**Figure S1** Principal Coordinate analysis (PCoA) of bacterial communities in activated sludge samples across different PWWTP treatment processes (A/O and O). (a) and different wastewater compositions (P-94, P-440, and P-450) (b).

**Figure S2** Microbial composition in the activated sludge at class level across different influent compositions (a) and different PWWTP treatment processes (b). The top 14 classes were shown, with the remaining classified as the “Others”.

**Figure S3** The LDA histogram shows taxa with LDA scores greater than 4.5 (a). The LEfSe cladogram shows bacterial biomarkers enriched in each treatment group (b). Circles from the center to the outside represent taxonomic levels from kingdom to species. The labels p, c, o, f, g, and s represent phylum, class, order, family, genus, and species, respectively. Circle diameter is proportional to relative abundance, and colored dots indicate taxa that differed significantly among samples. Species names on the right indicate biomarkers not shown in the figure, with corresponding letter-number codes matching the figure.

**Figure S4** The correlation between activated sludge microbial community composition and pollutant concentration in different PWWTP treatment processes. The width and the colour of the lines indicate the correlation coefficient and significance, respectively. The colour gradient represented the correlations of pairwise environmental factors.
